# THE ALTERNATIVE MODEL OF PERSONALITY DISORDERS: LONGITUDINAL PSYCHOMETRIC PROPERTIES IN OLDER ADULTS

**DOI:** 10.1093/geroni/igae098.2504

**Published:** 2024-12-31

**Authors:** Lisa Stone-Bury, Daniel L Segal

**Affiliations:** Bucknell University, United States; University of Colorado Colorado Springs, Colorado Springs, Colorado, United States

## Abstract

The Alternative Model of Personality Disorders (AMPD) is a dimensional model of personality disorders that contains two main diagnostic constructs: personality functioning and five pathological personality traits domains. This study examined the temporal stability and predictive validity of the AMPD’s two diagnostic constructs among older adults. It was hypothesized that (1) the AMPD will show high levels of both differential and absolute stability and (2) the AMPD domains measured at baseline will be significantly predictive of various salient aspects of psychosocial functioning at Time 2, including interpersonal, intrapersonal, health, and social functioning. Community-dwelling older adults (n = 200) were recruited online and completed self-report questionnaires of the AMPD and aspects of psychosocial functioning. A subsample of the original group (n = 172) completed the same questionnaires three months later. Correlations and paired samples t-tests partially supported the temporal stability of the AMPD: personality functioning demonstrated good temporal stability, whereas pathological personality traits were mixed (good differential stability and poor absolute stability). Hierarchical regressions supported the pathological personality trait model’s predictivity validity among older adults, with Time 1 scores significantly predicting personality, interpersonal, intrapersonal, health, and social functioning scores at Time 2. Overall, hypotheses were partially supported. Differential stability (relative positioning of a participant within the sample) of the AMPD’s pathological personality trait model appears questionable in older adults, which is notable given the brief period of time examined in the study. However, the AMPD showed good predictive validity, with the trait model predicting several clinically meaningful outcomes in later life.

